# Hierarchical Dynamic Bayesian Network-Based Fatigue Crack Propagation Modeling Considering Initial Defects

**DOI:** 10.3390/s22186777

**Published:** 2022-09-07

**Authors:** Yang Xu, Bin Zhu, Zheng Zhang, Jiahui Chen

**Affiliations:** 1 Key Lab of Smart Prevention and Mitigation of Civil Engineering Disasters of the Ministry of Industry and Information Technology, Harbin Institute of Technology, Harbin 150090, China; 2 Key Lab of Structures Dynamic Behaviour and Control of the Ministry of Education, Harbin Institute of Technology, Harbin 150090, China; 3 School of Civil Engineering, Harbin Institute of Technology, Harbin 150090, China; 4 China Railway Siyuan Survey and Design Group Co., Ltd., Wuhan 430063, China; 5 Hubei Key Laboratory of Track Security Service, Wuhan 430063, China

**Keywords:** fatigue crack propagation, hierarchical dynamic Bayesian network, multi-source uncertainty analysis, fracture mechanics, multi-scale finite element analysis, fatigue reliability, orthotropic steel decks

## Abstract

Orthotropic steel decks (OSDs) are inevitably subjected to fatigue damage caused by cycled vehicle loads in long-span bridges. This study establishes a probabilistic analysis framework integrating the dynamic Bayesian network (DBN) and fracture mechanics to model the fatigue crack propagation considering mutual correlations among multiple fatigue details. Both the observations of fatigue crack length from field inspection and monitoring data of vehicle loads from the weight-in-motion (WIM) system are utilized. First, fracture mechanics-based uncertainty analysis is performed to determine the multiple uncertainty sources in the Paris crack propagation model, material property, and observation data of crack length. The uncertainty of monitoring data of vehicle loads is ignored because of its high accuracy; consequently, the vehicle-load-related uncertainty is spontaneously ignored, which is also demonstrated to be very small on the investigated actual bridges. Second, a hierarchical DBN model is introduced to construct mutual dependencies among various uncertainties and intra-correlations in the propagation process of multiple fatigue cracks at different components. Third, a stochastic traffic model is established based on the WIM monitoring data and multi-scale finite element analysis via substructure techniques to determine the probability distribution of vehicle-load-related parameters. After variable discretization, a refined exact inference algorithm in a forward–backward–forward manner is adopted to estimate the posterior distribution of equivalent initial crack length and update the established DBN model. Finally, the proposed method is demonstrated by a numerical case study and a typical application on an actual cable-stayed bridge with steel box girders using OSDs in China. The results show that the probability distribution of equivalent initial crack size can be spontaneously derived with a larger mean value than the results of conventional empirical analysis. Furthermore, the component-level fatigue reliability is tracked and predicted based on the established crack propagation model.

## 1. Introduction

Orthotropic steel decks (OSDs) are widely used in long-span cable-stayed and suspension bridges, benefiting from the advantages of light weight, high strength, and quick assembling ability. Nevertheless, OSDs are frequently subjected to cycled vehicle loads and inevitably suffer from fatigue damage, significantly reducing the fatigue reliability and weakening the serviceability of bridges.

The fatigue crack propagation (FCP) process often develops from minor initial material flaws and manufactural defects, containing various uncertainty sources and bringing significant challenges for the accurate prediction of fatigue life. Conventional analysis approaches based on S-N curves and fracture mechanics are the most widely used methods for fatigue life assessment. The S-N curve-based approach describes the fatigue state and analyses the fatigue reliability according to various damage accumulation rules. It determines the fatigue damage state by conducting multiple stress-controlled fatigue tests as

(1) $$N=k\cdot \Delta S^{-m}\Leftrightarrow \log N=\log k-m\log\Delta S$$

where *N* denotes the number of cycles until failure, ∆*S* denotes the stress range of fatigue load, and *k* and *m* are two material-dependent constants.

It can be found in Equation (1) that the S-N curve only considers the effect of stress amplitude on fatigue life and could not reflect the process of fatigue crack propagation because no related parameters of crack size are considered. In addition, the S-N curve-based approach could not utilize the inspection data of fatigue crack size at different time steps to update the model parameter and fatigue state. The fracture mechanics-based approach supported by the Paris law [1] concentrates on FCP from the initial size of material flaws to the final critical crack size. Because S-N curve-based methods require plenty of fatigue experiments and are resource-expensive, this study investigates the FCP process based on the prior probability distribution of initial crack size and fracture mechanics. The field inspection data of fatigue crack size is also utilized to obtain the updated posterior probability distribution of initial crack size.

The first challenge for accurate fatigue life analysis based on fracture mechanics is determining the initial crack size or probability distribution for the concerned fatigue detail. Previous studies have shown that the initial crack size significantly influences the fatigue life and fatigue reliability of welding joints [2,3]. It is common sense that the crack initiation of welding structures would take a negligible time due to numerous existing material flaws and welding defects [4], including slag inclusion and incomplete penetration [5]. This study considers the effect of equivalent initial crack size (EICS) for the fatigue crack, which is an indicator of the welding quality and is also utilized to facilitate fracture mechanics-based fatigue analysis. Yang et al. [6] proposed a probabilistic framework for FCP to manage ship structures, in which the EICS was assumed to follow a normal distribution with a mean value of 0.5 mm and a variance of 0.1 mm^2^. Cui et al. [7] investigated the fatigue life of steel truss bridges considering the effects of residual welding stress, and an initial crack length of 0.15 mm was adopted for validation. In addition to directly using empirically assumed values, Bokalrud and Karlsen [8] investigated actual welding joints of ship structures and reported that the initial crack size followed an exponential distribution with a mean value of 0.11 mm. Moan et al. [9] performed over 4000 non-destructive tests on tubular joints and concluded that the mean value of initial crack size was 0.94 mm based on the exponential distribution assumption.

In summary, various initial crack sizes have been assumed in previous studies and are generally distributed within 0.1 mm–1 mm for welding joints in steel structures. Most existing methods empirically determined the initial crack size, and it was challenging to validate its reliability and accuracy. In addition, the inspection data of crack size were always ignored to update the initial crack size. Because the propagation speed in the crack initiation stage is very slow, the fatigue life and reliability would be determined with significant estimation errors using a wrongly assumed initial crack size. As a comparison, the proposed method can update the prior probability distribution of initial crack size based on the inspection data of crack size at different spatial locations and time steps.

Another challenge for the fracture mechanics-based approach is dealing with various uncertainty sources in the FCP process. Four categories of uncertainties in material, vehicle load, measurement data, and fracture mechanics model may exist. Conventional fracture mechanics-based approaches are deterministic and cannot deal with the randomness in the FCP process. According to previous studies [10,11], the model uncertainty of fracture mechanics and material uncertainty are recommended to be considered in the FCP process. Therefore, this study investigates the material uncertainty caused by the natural deviancies in the manufacturing process and the model uncertainty as the system error. Material uncertainty corresponds to the variability of the material-related parameter in the Paris law and the initial crack size. The variability of other material properties, including elastic modulus, shear modulus, and Poisson ratio, is less significant and assumed to be deterministic in this study. The uncertainty of vehicle load is another critical factor, and the cycled vehicle loads passing through a bridge is the principal source of fatigue degradation for welding joints. This study establishes a stochastic traffic model based on the monitoring data from the weigh-in-motion (WIM) system. Four traffic-related variables are considered, including vehicle types, gross weight, coefficients of axle weight, and annual traffic volume. Investigations reveal that the vehicle-load-related uncertainty can be spontaneously ignored because it is demonstrated to be very small in actual bridges. The measurement error of visual inspection data is considered and utilized as a random error term, while the uncertainty of monitoring data in the WIM system is ignored because of the data’s high accuracy. In addition, the model uncertainty from the Paris law and finite element model (FEM) is also considered in this study. Considering the abovementioned multiple categories of uncertainty, this study aims to establish a framework for ECIS estimation and fatigue reliability evaluation for OSDs considering the effects of equivalent initial defects.

Recently, dynamic Bayesian networks (DBNs) [12], considering diverse sources of uncertainty, have been adopted for structural deterioration modeling. DBN is the extension version of Bayesian networks (BNs) in the time dimension, comprising a series of BNs connected by directed edges [13]. It is particularly suitable for deterioration modeling because it facilitates the evaluation of the evolving states of time-dependent systems. Straub [14] developed a fundamental framework based on DBN to describe stochastic deterioration processes and predict fatigue crack growth. Zhu and Collette [15] presented a novel dynamic discretization method to improve the accuracy and robustness of DBN inference. Li et al. [16] proposed a DBN-based approach for tracking the health state of an aircraft wing, in which the diagnosis and prognosis tasks were accomplished by the particle filtering algorithm. Chen et al. [17] established a DBN-based framework for monitoring and predicting the condition of the subsea pipeline considering the effects of corrosion.

In addition to the above component-level applications (correlations among different components are ignored), investigations on the system level of the whole structural system have also been performed. The hierarchical model has been utilized to express the correlations among variables following the same distribution based on identical influencing factors [18,19]. Schneider et al. [20] presented a DBN-based approach to track and update the deterioration state of a concrete bridge in terms of rebar corrosion, in which hyperparameters were introduced to model the spatial correlations of the corrosion process in different areas. Luque and Straub [21] proposed a hierarchical model to consider the correlation and dependency among the deterioration processes of different components in the system.

Despite the high dependence on subjective judgment and low stability of field inspection results, it is still regularly conducted for long-span cable-stayed bridges to detect structural damage and ensure structural safety. Measurement data of structural damage, including the spatial location, number, length, and width of fatigue cracks on each component, can be obtained by field inspections and structural health monitoring systems. For example, the local strain is sensitive to fatigue cracks and can be used as an indicator of fatigue damage. Strain transfer theory has been developed to establish the quantitative relationship between the sensing fiber and the host material to correct the measurement error and improve the measurement accuracy. A systematic review of strain transfer theory on measurement accuracy, design, and calibration has been summarized for optical fiber-based sensing in civil engineering [22]. A six-parameter expression of the damage ratio variable was proposed considering the effect of both the Lode angle and the hydrostatic pressure to expand the application scope of the existing damage ratio strength theory [23]. OSDs contain several types of similar fatigue details, and the inspection results of fatigue details at different locations may have strong correlations regarding the similar component geometry and vehicle load [24]. Given inspection data, DBN incorporated with the hierarchical model facilitates the updating of fatigue reliability at both the component and system levels. Heng et al. [25] adopted the hierarchical model proposed in [21] to evaluate the fatigue reliability of OSDs, in which the stochastic traffic model and FEM were combined to obtain the stress spectrum.

This study proposes a hierarchical DBN-based framework for ECIS estimation and fatigue reliability analysis, considering equivalent initial defects to investigate the fatigue deterioration process of OSDs in bridges. A hierarchical DBN model formulated by fracture mechanics is established to integrate various sources of uncertainties (including the mechanical deterioration model, material property, and inspection data). After the corresponding variables are discretized in the established DBN model, a refined exact inference algorithm is applied to derive the posterior distribution of EICS and predict the FCP process. A multi-scale FEM using substructure techniques is built to calculate the time history of vehicle-induced nominal stress at critical locations. In combination with Monte Carlo simulation (MCS), a stochastic traffic flow model is established to derive the probability distribution of vehicle-load-related parameters.

Three main tasks are accomplished using the proposed method in this study: (1) deriving the posterior distribution of EICS by updating the prior distribution recommended by BS7910 [26], (2) tracking and predicting the fatigue reliability according to the updated FCP process, (3) and demonstrating the effectiveness of the established framework for EICS estimation and fatigue reliability evaluation using the inspection data of 600 fatigue details at 10 time-points on an actual cable-stayed bridge in China.

The remainder of this paper is organized as follows. Section 2 presents the architecture of the established hierarchical DBN model and details of the refined exact inference algorithm. Section 3 introduces the implementation details of the stochastic traffic flow and multi-scale FEM using the monitoring data of the WIM system. The proposed method is verified in Section 4 by a numerical case study and a real-world application on an actual cable-stayed bridge. Finally, Section 5 concludes this paper.

## 2. Hierarchical Dynamic Bayesian Network for Fatigue Crack Propagation (FCP)

### 2.1. Fracture Mechanics-Based Multi-Source Uncertainty Analysis

In this study, the fracture mechanics-based crack propagation process is expressed by the Paris equation as

(2) $$\frac{da}{dN}=C{\left(\Delta K\right)}^{m}$$

where *a* denotes the crack length, *N* denotes the number of stress cycles. The material parameter *C* is generally determined by fatigue experiments; *m* is recommended as a constant by BS7910 [26]. ∆*K* denotes the range of stress intensity factor (SIF); *da*/*dN* denotes the crack propagation rate, and its value equals to the increment of crack length within a stress cycle.

The calculation method of SIF can be summarized into two categories: (1) direct approach—building a fine-meshed FEM of the crack tip and calculating the SIF based on numerical methods; (2) indirect approach—building a FEM without considering the crack geometry to calculate the far-field nominal stress at the crack and adopting an analytical formula to compute the SIF [27,28]. The direct approach is more complex and computationally expensive than the indirect one because the finite element analysis must be run numerous times when performing crack propagation analysis considering various uncertainties. Therefore, the indirect approach is utilized in this study for computational efficiency.

The range of SIF is formulated as

(3) $$\Delta K=G\Delta S\sqrt{\pi a}$$

where ∆*S* denotes the nominal stress range, and *G* denotes the geometric factor related to the crack length *a*. In this study, the uncertainty of *G* is considered by introducing a random variable *F* multiplying with a constant value of $\bar{G}$. Thus, Equation (2) is transformed as

(4) $$\Delta K=\bar{G}F\Delta S\sqrt{\pi a}$$

Based on Equations (2) and (4), the crack length after the current time step can be derived by

(5) $$\begin{array}{l} \frac{da}{dN}=C{\left(\Delta K\right)}^{m}\Leftrightarrow da=C{\left(\Delta K\right)}^{m}dN\Rightarrow \Delta a=C{\left(\Delta K\right)}^{m}\Delta N,\Delta K=\bar{G}F\Delta S\sqrt{\pi a}\\ a_{t+1}=a_{t}+\Delta a_{t}\approx a_{t}+{\left(\bar{G}\sqrt{\pi }\right)}^{m}\cdot C{\left(F\sqrt{a_{t}}\right)}^{m}\cdot \Delta N\Delta S^{m} \end{array}$$

where ∆*N*∆*S^m^* is related to the vehicle load within the current time step. In this study, a single variable *J* = ∆*N*∆*S^m^* is utilized to consider the influence of vehicle loads for simplification. A stochastic traffic flow model integrated with Monte-Carlo simulation is designed to compute the probability distribution of *J*. Note that the vehicle-load-related uncertainty is ignored in this study because the variability of *J* is demonstrated to be very small in actual bridges (*J* is regarded as a constant using its mean value). Details will be given in Section 3.2. Thus, Equation (5) can be written as

(6) $$a_{t+1}=a_{t}+J{\left(\bar{G}\sqrt{\pi }\right)}^{m}C{\left(F\sqrt{a_{t}}\right)}^{m}$$

Next, the uncertainty of measurement data is considered. Two categories of measurement data are utilized in this study: field inspection data of the fatigue crack length in OSDs and monitoring data of vehicle loads by the WIM system. The visual inspection data of crack length is assumed to follow a zero-mean Gaussian distribution with a standard deviation *σ_obs_*. Thus, the inspection data of fatigue crack length can be expressed as

(7) $$a_{t}^{obs}=a_{t}+\varepsilon_{obs},\varepsilon_{obs}∼N\left(0,\sigma_{obs}{}^{2}\right)$$

It should be noted that the proposed probabilistic framework can also deal with other types of distributions for measurement error. The uncertainty of the monitoring data of vehicle loads is ignored because of the high accuracy of the WIM system.

Therefore, the uncertainty in the FCP process can be expressed by the Paris-model-related random variable *F*, material-related random variable *C*, and variability of observation data of crack length $a_{t}^{obs}$.

### 2.2. Hierarchical Dynamic Bayesian Network for FCP of OSD System

Following the above uncertainty analysis, the fatigue deterioration of OSDs is highly correlated at different structural components due to the similar welding quality, material property, and vehicle load. Therefore, three types of correlations are considered in this study: (1) correlation of the fracture-mechanics-model parameter *F*; (2) correlation of the material parameter *C* in the Paris law; (3) correlation of the EICS *a*_0_ ($a_{t}{|}_{t=0}$) at different locations of fatigue details (mainly affected by the welding quality). Variables representing these correlations are assumed to be equally correlated and follow identical marginal distributions.

**Figure 1:**
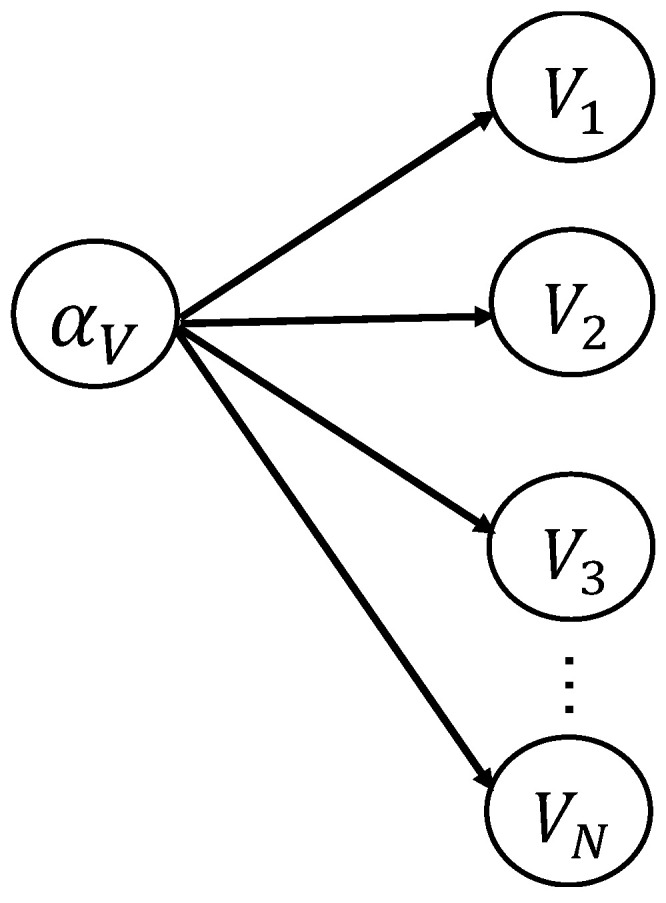
Schematic of the hierarchical model with a parent-node hyperparameter.

As shown in Figure 1, a shared uncertainty hyperparameter $\alpha_{V}$ is introduced as the parent node to statistically interrelate the child-node random variables *V*_1_, ⋯ *V_N_*. Here, *V* is a representative variable for the considered category of uncertainty, which can denote *F* for the model uncertainty, *C* for the material uncertainty, and *a*_0_ for the uncertainty in EICS. *N* denotes the number of considered individuals (here, it equals to the number of structural components in the OSD system). The uncertainty of $\alpha_{V}$ leads to the fact that variables $V_{1},\cdots V_{N}$ are statistically dependent according to the D-separation rule [13] in the theory of Bayesian networks and causal inference.

In this study, the hyperparameter $\alpha_{V}$ is assumed to follow the standard normal distribution. Meanwhile, each child-node variable is assumed to be conditionally related on the parent-node hyperparameter $\alpha_{V}$ as

(8) $$F_{V|\alpha_{V}}\left(V_{i}\right)=\Phi \left(\frac{\Phi^{-1}\left(F_{V}\left(V_{i}\right)\right)-\sqrt{\rho_{X}}\cdot \alpha_{V}}{\sqrt{1-\rho_{X}}}\right)$$

where $V_{i}$ denotes the *i*th random variable of $V_{1},\cdots V_{N}$, $F\left(V_{i}\right)$ denotes the cumulative distribution function, Φ denotes the standard normal distribution, $\rho_{X}$ denotes the correlation coefficient in the standard normal space. The correlation coefficient between arbitrary two child-node variables in $V_{1}\cdots V_{N}$ is assumed to be identical and noted as $\rho_{V}$, following the same marginal distribution $F_{V}$. $\rho_{X}$ can be estimated by $\rho_{V}$ and $F_{V}$ using numerical methods proposed in previous studies [29,30], which is not the focus of this study and thus omitted here.

**Figure 2:**
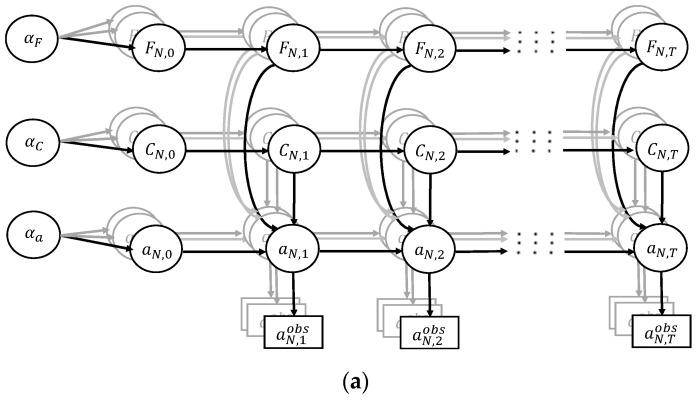

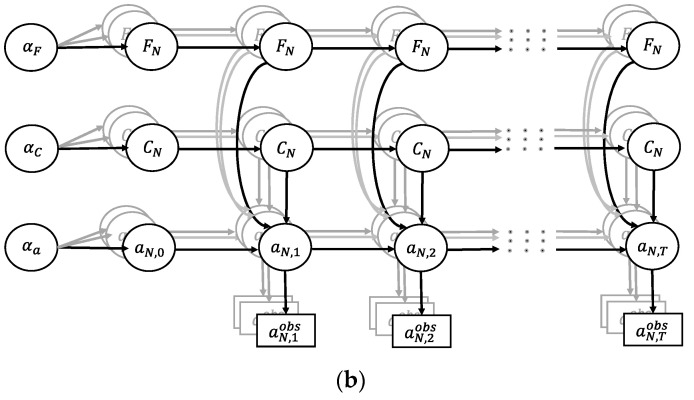
Schematic of established dynamic Bayesian network for FCP in OSD system. (**a**) General framework with time-dependent model uncertainty and material uncertainty. (**b**) Specific diagram with time-independent model uncertainty and material uncertainty in this study.

Figure 2 shows the overall schematic of the established hierarchical DBN to model the system-level FCP in OSDs. The above fracture mechanics model-related, material-related, and EICS-related uncertainties are expressed by the corresponding nodes in each row for *F*, *C*, and *a*, respectively. The first and second subscripts denote the component number and the time step, respectively. For instance, $a_{i,0}$ denotes the EICS of the *i*th component. A three-tuple hyperparameter group $\left[\alpha_{F},\alpha_{C},\alpha_{a}\right]$ are introduced as different parent nodes to model the correlations among different components (stacked from 1 to *N*) in the hierarchical model. The observation data for fatigue crack length of the *i*th component at time step *t* is also considered by $a_{i,t}^{obs}$.

The established hierarchical DBN model discretizes the continuous deterioration process of FCP, and each column refers to the evolution of the fatigue crack length at a specific time step. The effects of crack increment on SIF during each time step are ignored, and the corresponding SIF is regarded as a constant before the next step. In addition, a commonsensical assumption is adopted: that the model uncertainty and material uncertainty represented by random variables *F* and *C* do not evolve over time and are considered time-independent, i.e., $F_{i,t}=F_{i,t-1},C_{i,t}=C_{i,t-1},t=1\cdots T$. Figure 2a shows the general framework of the dynamic Bayesian network with time-dependent *F* and *C*. Figure 2b shows the specific diagram established in this study under consistent model uncertainty and material uncertainty with time-independent *F* and *C*. In this study, the general framework of the dynamic Bayesian network in Figure 2a is simplified to Figure 2b with the time-independent model uncertainty and material uncertainty.

### 2.3. Refined Exact Inference Algorithm for Dynamic Bayesian Network Updating

Two types of inference algorithms for DBN have been developed to deal with continuous and discrete variables [12], respectively. However, algorithms for continuous variables are generally sampling-based, leading to low computational efficiency when the number of nodes increases. Additional samples are further required for failure reliability analysis, which will inevitably increase the computation cost.

To cope with this issue, the sequential discrete procedure is adopted to determine the conditional probability tables (CPTs) of nodes in the established hierarchical DBN model. Note that the computational complexity is proportional to the product of the number of states of the pre-designed hyperparameters. Therefore, it is a trade-off between computational efficiency and the accuracy of correlation modeling. Table 1 summarizes the discretization schemes of random variables in the established hierarchical DBN model. $N_{\alpha },\;N_{F},\;N_{C},N_{a}$ denote the number of states for different random variables of parent-node hyperparameter α, fracture mechanics model-related parameter *F*, material property-related parameter *C*, and crack length-related parameter *a*, respectively. *A* and *B* represent the second-left and second-right values of equally distributed intervals, respectively. The corresponding parameters in the discretization schemes are described in Section 3.3 of implementation details.

Since corresponding variables have been discretized in the established hierarchical DBN model, this study designs a refined exact inference algorithm with high computational efficiency following a forward–backward–forward propagation paradigm.

The forward inference process is conducted in a parallel way because nodes are only connected through the shared hyperparameters for different components, as shown in Figure 2. For the *i*th component, the conditional joint probability at time step *t* is calculated as

(9) pFi,t, Ci,t, ai,t|α,ai,1:tobs=pFi,t, Ci,t, ai,t|α,ai,1:t−1obspai,tobs|ai,tpai,tobs|α,ai,1:t−1obs

where *i* and *t* represent the index number of components and time steps, i=1⋯N, t=2⋯T.

The first term in the numerator of Equation (9) can be computed based on the conditional joint probability at the previous time step as

(10) $$p\left(F_{i,t},\;C_{i,t},\;a_{i,t}|\alpha ,a_{i,1:t-1}^{obs}\right)=\underset{a_{i,t-1}}{\sum }p\left(a_{i,t}|F_{i,t},\;C_{i,t},\;a_{i,t-1}\right)\underset{C_{i,t-1}}{\sum }p\left(C_{i,t}|C_{i,t-1}\right)\;\times \underset{F_{i,t-1}}{\sum }p\left(F_{i,t-1},\;C_{i,t-1},\;a_{i,t-1}|\alpha ,a_{i,1:t-1}^{obs}\right)$$

The above forward propagation algorithm can be conducted recursively from the second time step. The conditional joint probability at the initial time step is calculated as

(11) $$p\left(F_{i,0},C_{i,0},\;a_{i,0}|\alpha \right)\propto p\left(F_{i,0}|\alpha \right)p\left(C_{i,0}|\alpha \right)p\left(a_{i,0}|\alpha \right)$$

Note that all terms on the right side of Equation (11) could be determined based on the CPTs of DBN.

Equation (8) indicates that the probability distribution of EICS ($a_{i,0}$) is conditional on the corresponding hyperparameter $\alpha_{a}$. Therefore, the posterior probability distribution of hyperparameters $\alpha =\left[\alpha_{F},\alpha_{C},\alpha_{a}\right]$ should be first obtained to derive the posterior probability distribution of EICS. Next, the backward propagation is adopted to parallelly update the posterior distribution $p\left(\alpha |a_{1:N,1:t}^{obs}\right)$ of α given the observations by

(12) $$p\left(\alpha |a_{1:N,1:t}^{obs}\right)\propto p\left(\alpha |a_{1:N-1,1:t}^{obs}\right)\overset{t}{\underset{k=2}{\prod }}p(a_{N,k}^{obs}|\alpha ,a_{N,1:k-1}^{obs})$$

Note that the multiplication term at the right side of Equation (12) is the denominator of Equation (9). The posterior distribution of hyperparameters considering only one component is calculated as

(13) $$p\left(\alpha |a_{1,1:t}^{obs}\right)\propto p\left(\alpha \right)\overset{t}{\underset{k=2}{\prod }}p(a_{1,k}^{obs}|\alpha ,a_{1,1:k-1}^{obs})$$

where $p\left(\alpha \right)$ denotes the prior joint probability and can be calculated by the prior probability of independent hyperparameters as $p\left(\alpha \right)=p\left(\alpha_{F}\right)p\left(\alpha_{C}\right)p\left(\alpha_{a}\right)$. Based on the posterior probability distribution of the hyperparameters α, the posterior probability distribution of the crack size at any time step can be further deduced.

Given all the observations up to time step *t*, the posterior distribution of crack length at time step *t* can be derived as

(14) $$p\left(a_{i,t}|a_{1:N,1:t}^{obs}\right)=\underset{F_{i,t}}{\sum }\underset{C_{i,t}}{\sum }\underset{\alpha }{\sum }p\left(F_{i,t},C_{i,t},\;a_{i,t},\alpha |a_{1:N,1:t}^{obs}\right)$$

The multiplication term at the right side of Equation (14) is calculated as

(15) $$p\left(F_{i,t},C_{i,t},\;a_{i,t},\alpha |a_{1:N,1:t}^{obs}\right)=p\left(F_{i,t},C_{i,t},\;a_{i,t}|\alpha ,a_{i,1:t}^{obs}\right)p(\alpha |a_{1:N,1:t}^{obs})$$

The first term on the right side of Equation (15) can be iteratively calculated from Equation (9) to Equation (11), and the second term is the posterior probability distribution of the hyperparameters, which can be determined from Equations (12) and (13).

Given all the observations up to time step t, the posterior distribution of crack length at the past time step t−h can be derived as

(16) $$p\left(a_{i,t-h}|a_{1:N,1:t}^{obs}\right)=\underset{F_{i,t-h}}{\sum }\underset{C_{i,t-h}}{\sum }\underset{\alpha }{\sum }p\left(F_{i,t-h},C_{i,t-h},\;a_{i,t-h},\alpha |a_{1:N,1:t}^{obs}\right),\;0\leq h\leq t$$

where

(17) $$p\left(F_{i,t-h},C_{i,t-h},\;a_{i,t-h},\alpha |a_{1:N,1:t}^{obs}\right)=p\left(F_{i,t-h},C_{i,t-h},\;a_{i,t-h}|\alpha ,a_{i,1:t}^{obs}\right)p\left(\alpha |a_{1:N,1:t}^{obs}\right)$$

in which the first term on the right side can be obtained by

(18) $$\begin{array}{c} p\left(F_{i,t-h},C_{i,t-h},\;a_{i,t-h}|\alpha ,a_{i,1:t}^{obs}\right)\\ \;\;\;\;\;\;\;\propto p\left(F_{i,t-h},C_{i,t-h},\;a_{i,t-h}|\alpha ,a_{i,1:t-h}^{obs}\right)p\left(a_{i,t-h+1:t}^{obs}|F_{i,t-h},C_{i,t-h},\;a_{i,t-h}\right) \end{array}$$

The first term on the right side of Equation (18) can be calculated by the forward inference process, and the second term can be recursively calculated starting from

(19) $$p\left(a_{i,t+1:t}^{obs}|F_{i,t},C_{i,t},\;a_{i,t}\right)=1$$

Equation (19) indicates that the second term on the right side of Equation (18) degrades to 1 at h=0, because the existing observation ends with time step *t*. Finally, the posterior distribution of EICS can be obtained by setting h=t.

For fracture mechanics-based reliability analysis, the fatigue failure is generally defined until the crack length reaches a specific value (named as the critical crack length $a_{critial}$). To this end, the limit state function could be established as

(20) $$g_{i}\left(a_{i.t},t\right)=a_{critical}-a_{i,t}$$

where $g_{i}$ denotes the limit state function for reliability analysis of the *i*th component.

## 3. Implementation Details

### 3.1. Stochastic Traffic Flow Model

In this section, a stochastic traffic flow model is established based on the monitoring data of vehicle loads from the WIM system, after which the finite element analysis is performed to derive the time histories of vehicle-induced stress.

A highway two-way six-lane cable-stayed bridge with a total length of 964 m and a main span of 460 m in China is employed in this study. Seven typical types of vehicles are extracted from the monitoring data of the WIM system, which are shown in Table 2.

**Figure 3:**
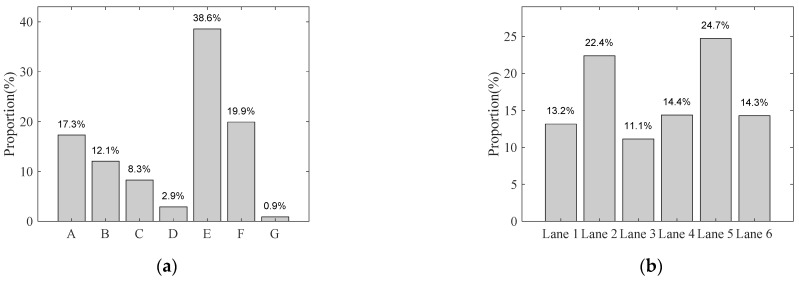
Statistical proportions of traffic flow in the investigated bridge. (**a**) Proportion of different vehicle types. (**b**) Proportion of traffic flow for each lane.

In this study, four variables are utilized to describe the stochastic traffic flow: (1) vehicle types, (2) gross vehicle weight, (3) coefficients of axle weight, and (4) annual traffic volume. Figure 3a shows the statistical proportions of the number of vehicles for different types, and Figure 3b shows the corresponding proportion in each lane.

**Figure 4:**
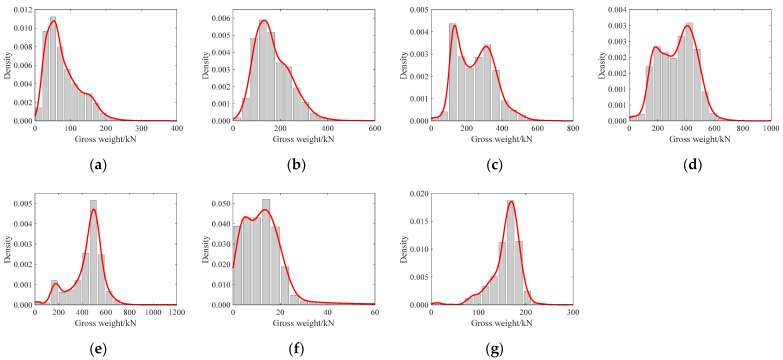
Probability distribution of gross weight for each type of vehicle. (**a**) Type A. (**b**) Type B. (**c**) Type C. (**d**) Type D. (**e**) Type E. (**f**) Type F. (**g**) Type G.

The frequency histograms of the gross weight of each vehicle type and the corresponding estimated probability density functions (PDFs) are shown in Figure 4. The following analysis ignores the F-type vehicles due to their lightest weights.

The axle weight fits the best with normal distribution. Nevertheless, to avoid excessive finite element analysis, the variability of axle weight ratio is ignored when generating random vehicle samples in this study. The mean values of the axle weight ratio are listed in Table 3, in which the F-type vehicle is omitted.

**Figure 5:**
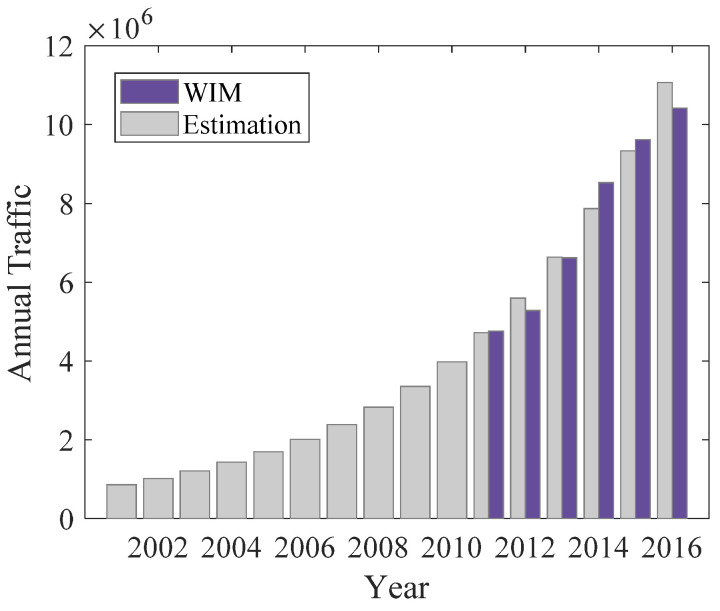
Comparison of monitoring and estimated annual average traffic volume.

The investigated bridge in this study was opened to traffic in 2001. However, the structural health monitoring system was installed until 2011, indicating that the traffic flow between 2001 and 2011 was not monitored. To cope with this problem, the average annual daily traffic growth model proposed by Šliupas [31] is adopted to estimate the traffic volume during the unmonitored period as

(21) $$lnV_{t}=b+k\times t$$

where $V_{t}$ denotes the average annual daily traffic volume observed at the *t*th year, *k* and *b* are constants obtained by linear regression. The estimated traffic volumes for different years are shown in Figure 5, which shows a good agreement with the monitoring results.

**Figure 6:**
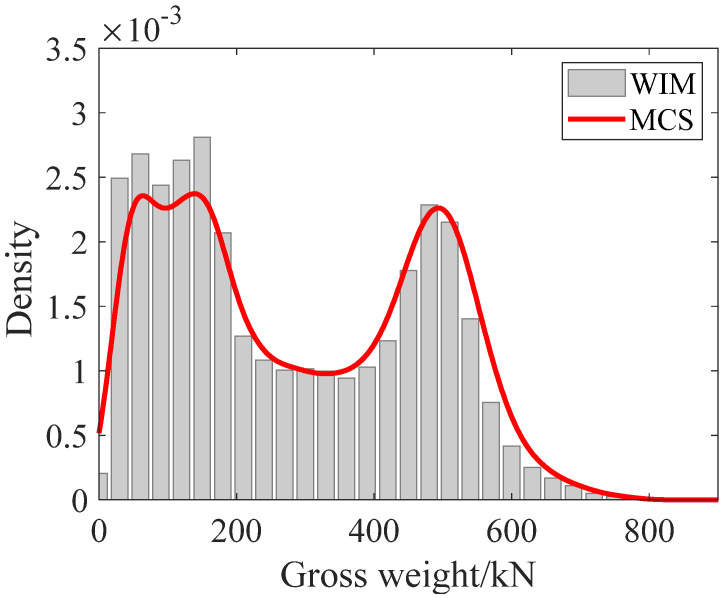
Comparison of monitoring and simulated probability distribution of gross vehicle weight.

To verify the above stochastic traffic flow model, the probability distribution of gross vehicle weight of 90,000 vehicles from the WIM system is compared with the simulated results using MCS, as shown in Figure 6. The simulated probability distribution of gross vehicle weight agrees reasonably well with the actual monitoring results, which demonstrates the efficiency of the established stochastic traffic model.

### 3.2. Multi-Scale Finite Element Analysis

**Figure 7:**
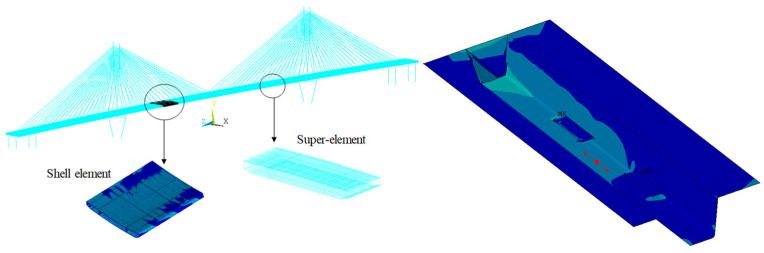
Multi-scale finite element model and nominal stress extraction of the investigated long-span cable-stayed bridge.

After the stochastic traffic flow is modeled, a multi-scale FEM of the investigated long-span cable-stayed bridge is built via the substructure technique to calculate the time history of vehicle-induced nominal stress, which is particularly effective for large and complex structures. The degree of freedom of the bridge system can be significantly reduced without the loss of accuracy. As shown in Figure 7, the girder is modeled by the shell element and super element (condensed from a group of shell elements). The tensile stress along the bridge at the surface of the critical location is extracted as the nominal stress to calculate SIF.

**Figure 8:**
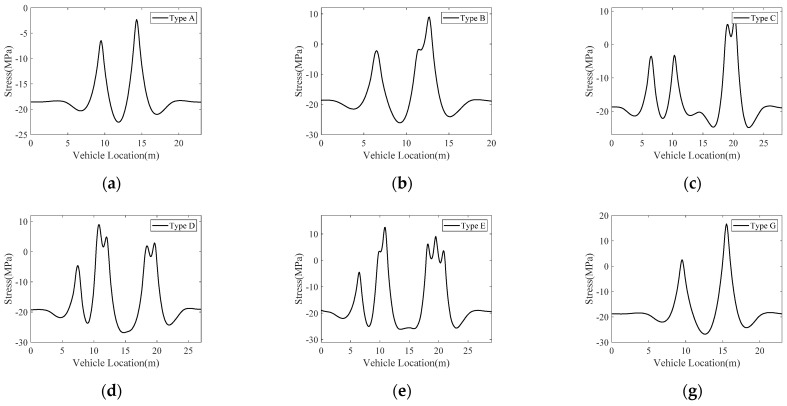
Time histories of vehicle-induced nominal stress at the critical location for different vehicle types. (**a**) Type A. (**b**) Type B. (**c**) Type C. (**d**) Type D. (**e**) Type E. (**g**) Type G.

The time histories of vehicle-induced nominal stress at the critical location for various types of vehicles are shown in Figure 8, in which the F-type vehicle is omitted. Note that all the calculated nominal stresses at the initial time are negative, which indicates they are compression stresses. This phenomenon is caused by the dead load of the cable-stayed bridge itself. The plates inside OSDs were welded to the U-ribs after the bridge was constructed; therefore, compression stresses induced by the dead weight should be eliminated in this study, and the initial stresses should be assigned to zero.

In addition, previous studies have shown that the residual welding stress at welded toes may oscillate around the material yield strength [7], which results in the aggravation of fatigue cracking. Therefore, the nominal stress range is defined as the gap between the maximum and minimum values inside one stress cycle in this study, which can be obtained from the time history of vehicle-induced nominal stress at the critical location by the rain-flow counting algorithm [32]. Note that vehicular interactions in the traffic lane are ignored in this study due to the short effective stress influence line on OSDs.

As stated in Section 2.1, the vehicle-related term $J=\Delta N\Delta S^{m}$ is adopted to simplify the FCP model. It relates to the number of vehicles (ΔV) within a time step, which is set as ∆*V* = 20,000 in this study.

For each type of vehicle, the vehicle-related term is calculated as

(22) $$J_{x}=\overset{N_{x}}{\underset{i=1}{\sum }}\Delta S_{x,i}^{m}n_{x,i}$$

where *x* denotes the vehicle type, i.e., A, B, C, D, E, F (ignored as stated before), and G. $N_{x}$ denotes the number of stress amplitudes obtained by rain-flow counting in the time history shown in Figure 8. $\Delta S_{x}$ denotes the stress amplitude for the *x*-type vehicle, and $n_{x,i}$ denotes the corresponding stress cycles.

The probability distribution of *J* is determined based on MCS as

(23) $$J=\overset{NV_{A}}{\underset{i=1}{\sum }}J_{A}{\left(\frac{w_{i}}{W_{A}}\right)}^{m}+\overset{NV_{B}}{\underset{i=1}{\sum }}J_{B}{\left(\frac{w_{i}}{W_{B}}\right)}^{m}+\cdots +\overset{NV_{G}}{\underset{i=1}{\sum }}J_{G}{\left(\frac{w_{i}}{W_{G}}\right)}^{m}$$

where $NV_{x}$ denotes the number of *x*-type vehicles obtained by MCS, $W_{x}$ denotes the gross vehicle weight utilized in calculating the time history of nominal stress for the *x*-type vehicles, $w_{i}$ denotes the simulated gross vehicle weight.

**Figure 9:**
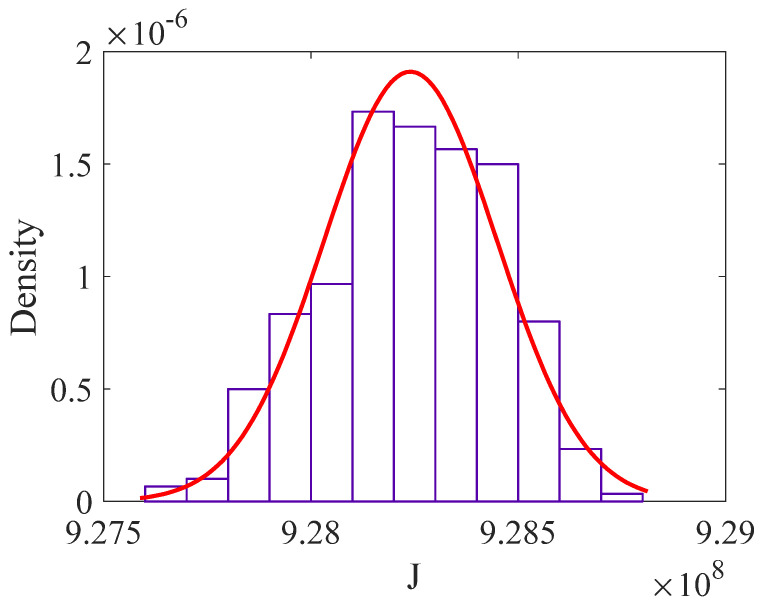
Probability distribution of the vehicle-related parameter *J*.

After 300 iterations of MCS, the histogram and fitted PDF of *J* are shown in Figure 9, indicating that *J* follows the normal distribution. Note that the variability of *J* is relatively small (the coefficient of variation is only 2.249 × 10^−4^); hence, it is acceptable to be a deterministic value. Therefore, the mean value of *J* is adopted in this study as $J=\mu_{J}=9.282\times 10^{8}$.

### 3.3. Parameter Setups in Hierarchical DBN for FCP Analysis

Parameters in the discretization schemes of random variables include the number of states and interval boundaries of different random variables for parent-node hyperparameter α, fracture mechanics model-related parameter *F*, material property-related parameter *C*, and crack length-related parameter *a*.

Table 4 shows the variable description and parameter setups of probability distribution in the established hierarchical DBN model. The material parameter *m* in the Paris law is set as a constant of 2.8 according to BS7910 [26]. The average geometric factor $\bar{G}$ is set as a constant of 1.2.

The correlation coefficients of fracture mechanics model-related parameter *F*, material property-related parameter *C*, and crack length-related parameter *a* among different components are equally defined as $\rho_{F}=\rho_{C}=\rho_{a}=0.8$. Detailed setups of parameters in Table 1 are reported as follows: *N_α_* = 5, *N_F_* = *N_C_* = 30, *N_a_* = 80, *A_α_* = 0.2, *B_α_* = 0.8, *A_F_* = 0.5, *B_F_ =* 1.5, *A_c_* = 3.18 × 10^−13^, *B_α_* = 2.34 × 10^−12^, *A_a_* = In (0.01), *B_a_* = In (70). In this study, the critical crack length is set to half of the width of the U-rib bottom in OSDs of the steel box girder, i.e., $a_{critical}=70\;\mathrm{mm}$.

## 4. Results and Discussion

### 4.1. Demonstration by Numerical Simulations

A numerical case study is performed in this study to validate the effectiveness of the established hierarchical DBN model for FCP analysis. Then, the refined exact inference algorithm is applied in a forward–backward–forward manner to derive the posterior distribution of EICS and update the hierarchical DBN model. The main procedures are summarized as follows.

**Figure 10:**
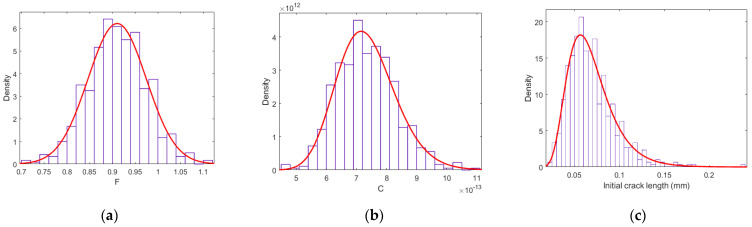
Probability distributions of fracture mechanics parameters: (**a**) model-related parameter *F*, (**b**) material property-related parameter *C*, and (**c**) initial crack length at the initial time step.

**Figure 11:**
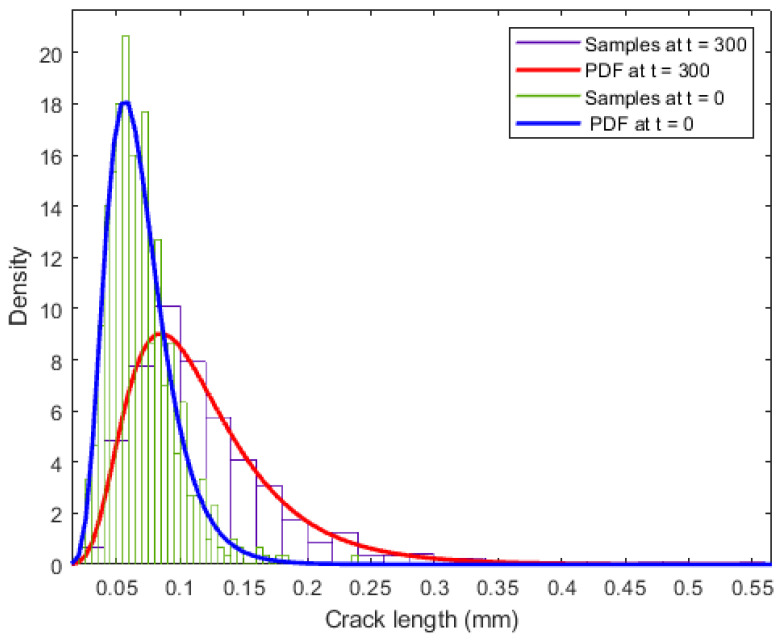
Comparison of probability distributions of crack length at *t* = 0 and *t* = 300.

(1) Based on the assumed distributions in Table 4, 600 samples are generated for each of $F_{i,0},\;C_{i,0},\;a_{i,0},\;i=1,2,\cdots ,600$, as shown in Figure 10.
(2) For each combination of $F_{i,0},\;C_{i,0},\;a_{i,0}$, FCP analysis is performed in a continuous space based on Equation (6). The comparison of the probability distribution of crack length at *t* = 0 and *t* = 300 is shown in Figure 11, which indicates a significant increase in crack length.
(3) Then, crack lengths under each combination at time steps *t* = 300:10:390 are predicted and transferred into the discrete states based on the discretization scheme shown in Table 1.
(4) The simulated states of different cracks at time steps *t* = 300:10:390 are utilized to update the prior probability distribution of parent-node hyperparameters and the EICS using the refined exact inference algorithm introduced in Section 2.3.

**Figure 12:**
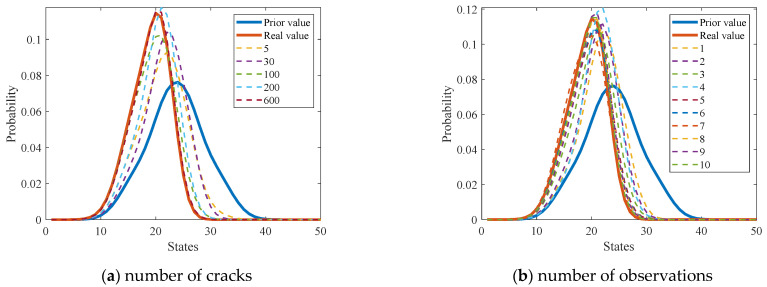
Probability distribution updating of initial crack length with the increasing number of (**a**) considered cracks and (**b**) adopted observations.

Figure 12a shows the updating process of the probability distribution of equivalent initial crack size (EICS). The results show that the posterior probability distribution gradually evolves as the number of cracks increases. It can be observed that the posterior probability distribution is very close to the actual one (with only a tiny variation) after more than 100 cracks are considered. Figure 12b shows that the posterior probability distribution also changes as the number of observations increases. It can be found that the posterior distribution is similar to the actual one when the number of observations exceeds six. In addition, numerical experiments demonstrate that using more temporal–spatial inspection data leads to a much more precise estimation of the probability distribution of the initial crack size. It demonstrates that considering the observation uncertainty of crack length enables the accurate estimation of the probability distribution for EICS.

The Hellinger distance is calculated as the quantitative evaluation metric of the estimation accuracy between the updated and ground-truth probability distributions of initial crack size as

(24) $$H(P,Q)=\sqrt{\frac{1}{2}\sum_{i}{\left(\sqrt{p_{i}}-\sqrt{q_{i}}\right)}^{2}}$$

where *H* denotes the Hellinger distance, *P* and *Q* denote the updated and ground-truth probability distributions of initial crack size, *i* denotes the index of discrete intervals, *p_i_* and *q_i_* denote the corresponding probability in the *i*th interval for distributions *P* and *Q*, $\sum_{i}p_{i}=\sum_{i}q_{i}=1$.

**Figure 13:**
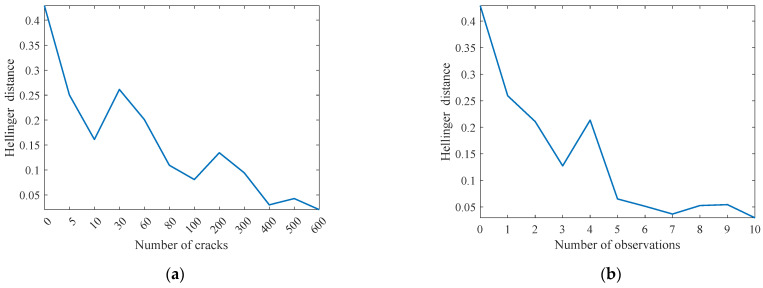
Hellinger distance variation between updated and ground-truth distributions of initial crack size using spatial–temporal inspection data. (**a**) Number of cracks. (**b**) Number of observations.

Figure 13 shows the Hellinger distance variation between the updated and ground-truth probability distributions of initial crack size considering the inspection data of various numbers of cracks and observations. The Hellinger distance with zero crack and zero observation corresponds to the assumption error between the prior and ground-truth probability distributions of initial crack size. The results show that using more spatial–temporal inspection data at different crack locations and time steps leads to a much more precise estimation of the initial crack size distribution with a lower Hellinger distance. It also indicates that using numerous inspection data of crack size to update the prior distribution of initial crack size can alleviate the random observation errors.

**Figure 14:**
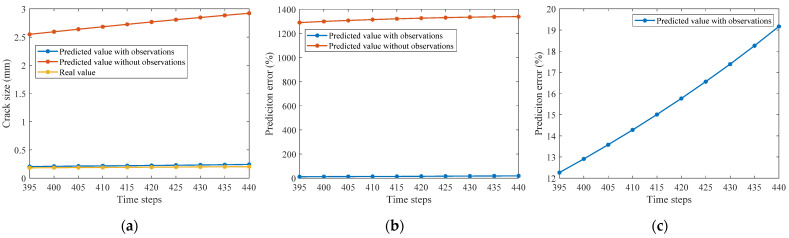
Comparisons of the prediction error of crack size with/without observation data. (**a**) Absolute prediction error. (**b**) Relative prediction error. (**c**) Zoom-in relative prediction error.

The fatigue crack size can be predicted based on Equations (5) and (6) at any future time step. However, the prediction error would explode due to the error accumulation. This study uses inspection data of crack size at various crack locations and time steps to update the prior probability distribution of initial crack size and reduce the prediction error. An additional numerical experiment is performed to predict 50 more time steps using the observation data of crack size in the 390 former time steps. Predicted crack sizes at every five steps are calculated using the observation data to update the established DBN model and compared with actual values and those without observation data, as shown in Figure 14. Although the prediction error increases with time, the relative prediction error is highly reduced by more than 60 times, from over 1200% to less than 20%. The predicted crack size using only prior information is much larger than the actual value, which suggests the effectiveness of using observation data to update the EICS and forecast the crack propagation by the proposed method. The results also indicate that the observation data is essential to updating the established DBN model for crack propagation prediction.

### 4.2. Demonstration by Actual Bridge Inspections

**Figure 15:**
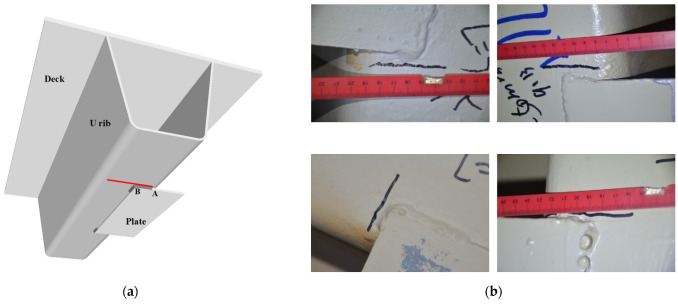
Actual bridge inspections for fatigue cracks at welding joints. (**a**) Illustrations of welding joint and fatigue crack location. (**b**) Representative fatigue cracks at local welding joints.

In this section, 11 actual bridge inspections were performed on the investigated cable-stayed bridge between September 2016 and September 2017. Some representative inspection results of 604 welding details are illustrated in Figure 15.

**Figure 16:**
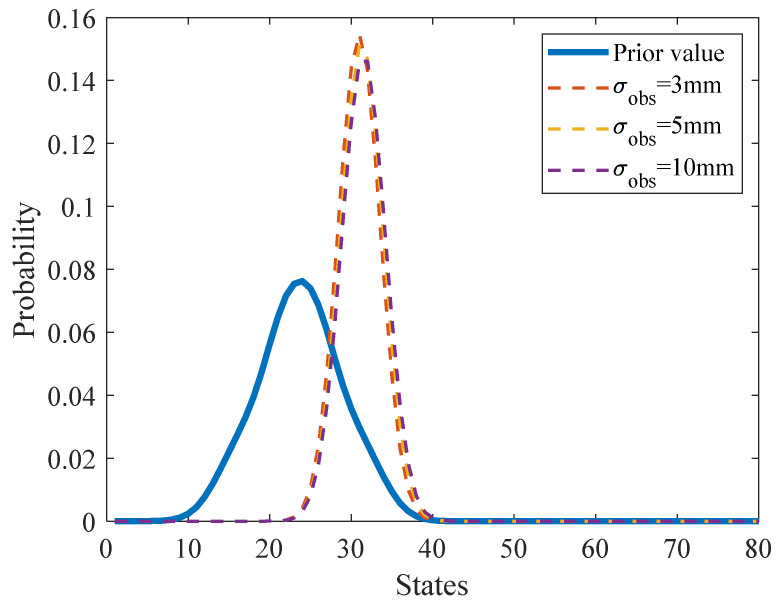
Posterior probability distribution of the EICS inferred from the field inspection results.

The observations of fatigue crack length are utilized to update the posterior distribution of EICS. Using the estimation of unmonitored traffic volumes (as illustrated in Section 3.1), the time steps corresponding to inspection actions can be determined. The corresponding parameter setups are defined as the same as the numerical case study in Section 4.1. Figure 16 shows the derived posterior distribution of EICS based on the field inspection observations of fatigue crack length with three different levels of observation standard deviations. The comparison shows that the posterior probability distribution of the EICS is almost the same under different observation errors, indicating that the established DBN model is robust to random observation errors. Furthermore, all the mean values of posterior probability distributions of the EICS under three levels of observation errors are larger than its prior probability distribution, indicating that the pre-assumed prior probability distribution of EICS is conservative.

**Figure 17:**
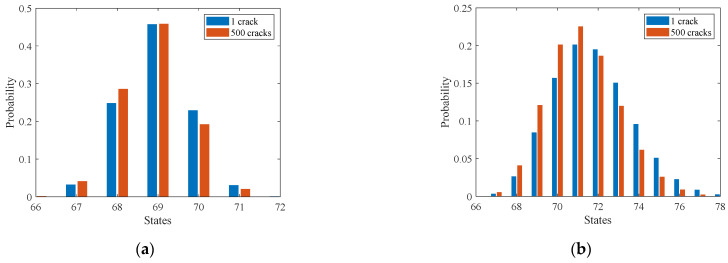
Comparison of posterior probability distributions of fatigue crack size considering the inspection data of only one crack and 500 cracks. (**a**) Fatigue crack size for current time step. (**b**) Fatigue crack size for future time step.

In addition, the current and future states of the concerned fatigue crack can be updated by the observation data of other cracks based on the hierarchical DBN model. As shown in Figure 17, after the inspection data of other cracks are involved, the posterior probability distributions of fatigue crack sizes at both current and future time steps obtain significant changes, indicating that the established DBN model constructs the dependencies and correlations among different fatigue cracks very well.

It should be noted that the initial crack size and its probability distribution under actual scenarios are difficult to measure in advance; therefore, we could not use the ground-truth distribution of initial crack size for comparison under real-world scenes. However, we performed numerical experiments in Section 4.1 to validate the accuracy of the updated probability distribution of initial crack size and demonstrate the effectiveness of the proposed method. The results show that the posterior probability distribution updated by the inspection data is very close to the ground truth.

The efficiency of the proposed DBN model is mainly determined by the number of states $N_{\alpha }$ for the parent-node hyperparameter ***α***. It is a trade-off issue because a larger state number leads to a more precise estimation with a longer computation time. Fortunately, visual inspections of fatigue cracks are usually performed every six months for long-span bridges with steel box girders, i.e., the established DBN model is updated by observation data every six months. The computation time cost of the proposed DBN model takes about 3~5 h to update all the fatigue cracks in the whole bridge on a workstation with an i7-10850 CPU, which is negligible compared with six months. Therefore, the efficiency of the proposed method is not emphatically investigated in this study.

### 4.3. Prediction of Component-Level Fatigue Reliability

**Figure 18:**
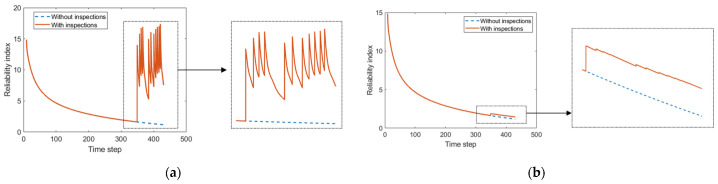
Reliability index changes of inspected and uninspected components considering the inspection data of fatigue cracks. (**a**) Inspected component. (**b**) Uninspected component.

The reliability index is defined as $\beta =-\Phi^{-1}\left(P_{f}\right)$, in which $P_{f}$ denotes the fatigue failure probability and can be calculated according to the limit state function of Equation (20). $\Phi^{-1}$ denotes the inverse cumulative distribution function of standard normal distribution. As shown in Figure 18a, the reliability index is updated according to the inspection results of fatigue crack length, which significantly differs from the reliability index without inspections. The reliability index increases after each inspection since the inspection data will lead to shorter EICS. Meanwhile, the deterioration rate (i.e., the gradient of the curve) suddenly increases after inspection. Observations of fatigue crack length also affect the reliability indexes of uninspected components due to the effective correlation modeling in the established hierarchical DBN model, as shown in Figure 18b.

Therefore, the fatigue state of different components can be interactively updated based on the established hierarchical DBN model and field inspection data of fatigue crack length. Note that the component-level reliability analysis framework can be extended to the system level by adopting a systematic scheme [25], which is not the focus of this study and is omitted here.

## 5. Conclusions

This study establishes a probability framework based on hierarchical DBN and fracture mechanics for fatigue crack propagation modeling considering initial defects in OSDs. The main conclusions are drawn as follows:

(1) Fracture mechanics-based uncertainty analysis is performed to determine uncertainty sources in the Paris crack propagation model, material property, and observation data of crack length. The uncertainty of monitoring data of vehicle loads is ignored because of the high accuracy of the WIM system. Actual applications also demonstrate that the vehicle-load-related uncertainty can be ignored.
(2) A hierarchical DBN model is introduced to construct mutual dependences and correlations among various uncertainties for fatigue crack propagation in different components. A refined exact inference algorithm is adopted to estimate the posterior distribution of EICS and update the DBN model in a forward–backward–forward manner. In addition, the component-level reliability index can be updated.
(3) A stochastic traffic model is established using the WIM monitoring data and multi-scale finite element analysis via substructure techniques. Numerical case studies and actual bridge inspections are performed to validate the effectiveness of the established hierarchical DBN model for FCP analysis.
(4) The results show that the established DBN model is robust to random observation errors and that the current and future states of the concerned fatigue crack can be updated by the observation data of other cracks based on the hierarchical DBN model, indicating that the proposed method constructs the dependencies and correlations among different fatigue cracks well.
(5) Numerical experiments demonstrate that using more temporal–spatial inspection data leads to a much more precise estimation of the probability distribution of the initial crack size. It also demonstrates that considering the observation uncertainty of crack length enables the accurate estimation of the probability distribution for EICS. The predictability of the proposed method on fatigue crack propagation forecast is also verified.

## Figures and Tables

**Table 1:** Discretization schemes of random variables in the established hierarchical DBN model.

Random Variable	Number of States	Interval Boundaries
$\alpha_{F},\alpha_{C},\alpha_{a}$	$N_{\alpha }$	$\left[-\infty ,\Phi^{-1}\left(A_{\alpha }:\frac{B_{\alpha }-A_{\alpha }}{N_{\alpha }-2}:B_{\alpha }\right),\infty \right]$
$F_{i,0},\;\;F_{i,t}$	$N_{F}$	$\left[0,\;A_{F}:\frac{B_{F}-A_{F}}{N_{F}-2}:B_{F},\;\infty \right]$
$C_{i,0},\;\;C_{i,t}$	$N_{C}$	$\left[0,\;A_{C}:\frac{B_{C}-A_{C}}{N_{C}-2}:B_{C},\;\infty \right]$
$a_{i,0},\;\;a_{i,t},\;a_{i,t}^{obs}$	$N_{a}$	$\left[0,\;\exp\left\{A_{a}:\frac{B_{a}-A_{a}}{N_{a}-2}:B_{a}\right\},\;\infty \right]$

**Table 2:** Typical vehicle types of the investigated cable-stayed bridge.

Vehicle Type	Truck Silhouette	Axle Span (m)
A	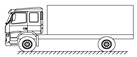	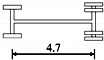
B	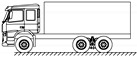	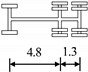
C	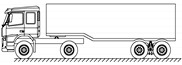	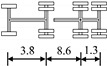
D	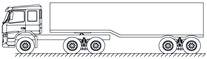	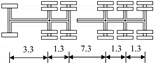
E	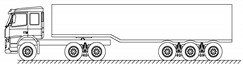	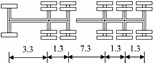
F	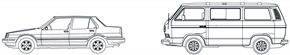	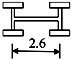
G	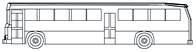	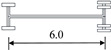

**Table 3:** Mean values of axle weight ratio for different vehicle types.

Vehicle Type	Mean Value of Axle Weight Ratio					
	Axle 1	Axle 2	Axle 3	Axle 4	Axle 5	Axle 6
A	0.392	0.608	-	-	-	-
B	0.264	0.266	0.470	-	-	-
C	0.185	0.207	0.291	0.317	-	-
D	0.154	0.274	0.199	0.184	0.190	-
E	0.109	0.138	0.204	0.193	0.178	0.177
G	0.392	0.608	-	-	-	-

**Table 4:** Variable description and distribution parameters in established hierarchical DBN model.

Variable	Description	Distribution Type	Mean Value	Standard Deviation
$a_{i,0}$	EICS	Lognormal	0.15	0.1
$J_{i}$	$J_{i}=\Delta N_{i}\Delta S_{i}{}^{m}$	Deterministic	9.282 × 10^8^	-
$lnC_{i,0}$	Material-related parameter	Normal	−27.76	0.23
$F_{i,0}$	Multiplier for crack geometric factor	Normal	1	0.1
$\alpha_{F},\alpha_{C},\alpha_{a}$	Hyperparameters of parent-nodes	Normal	0	1
$\sigma_{obs}$	Standard deviation of observation error	Deterministic	5	-

## Data Availability

The data presented in this study are available on request from the corresponding author. The data are not publicly available due to privacy.
